# Mapping Access to Nicotine Vaping Products for Smoking Cessation: Pharmacist Needs and Practice Implications in South‐East Melbourne

**DOI:** 10.1002/hpja.70213

**Published:** 2026-07-03

**Authors:** Isabella Papadopoulos, Melis Selamoglu, Wing Huen Nicolas Fu, May Foo, Zimo Wang, Nguyen Hoang, Millicent Castor, Sajal Saha, Alyce Jenkins, Chaturangi Yapa, Chris Barton

**Affiliations:** ^1^ Department of General Practice, School of Public Health and Preventive Medicine Monash University Clayton Victoria Australia; ^2^ Centre for Innovation in Infectious Diseases and Immunology Research (CIIDIR) Institute for Mental and Physical Health, School of Medicine, Deakin University Burwood Victoria Australia; ^3^ South East Public Health Unit (SEPHU) Monash Health Clayton Victoria Australia

**Keywords:** community pharmacy, e‐cigarettes, health equity, nicotine vaping products (NVPs), pharmacist practice, smoking cessation, spatial mapping, tobacco harm reduction

## Abstract

**Introduction:**

In October 2024, nicotine vaping products (NVPs) containing ≤ 20 mg/mL nicotine were reclassified to the Schedule 3 (‘pharmacist‐only’) category, permitting pharmacy‐based supply of unregistered therapeutic vapes for smoking cessation in Australia. This regulatory shift expanded pharmacists' roles in tobacco harm reduction, yet their preparedness and capacity to deliver counselling and product supply, specifically related to unregistered therapeutic vapes under the 2024 reforms, remain unclear. In South‐East Melbourne, where smoking and vaping rates are high, equitable access to cessation services via community pharmacy is a public health priority. This study examined the availability of therapeutic vapes in pharmacies and explored pharmacists' smoking cessation practices and support needs.

**Methods:**

A sequential mixed methods study was conducted across the South East Public Health Unit (SEPHU) catchment in Victoria. It included: (1) an audit of therapeutic vape availability in community pharmacies; (2) a survey of pharmacists' cessation practices, confidence and service provision and (3) qualitative interviews to contextualise findings. Survey and audit data were mapped using Geographic Information System (GIS) tools to examine spatial patterns in NVP availability, socioeconomic status (SES) and smoking and vaping prevalence. Interview transcripts were analysed using Braun and Clarke's 6‐step framework for thematic analysis. Data were collected between May and July 2025.

**Results:**

Of 418 community pharmacies audited in the catchment region, 174 (41.6%) reported supplying therapeutic vapes. Seventy (16.7%) pharmacists completed the survey; most (*n* = 30/32, 93%) dispensed therapeutic vapes fewer than five times weekly. Availability was higher in areas of greater socioeconomic advantage. Interviews revealed barriers to supply including perceptions of limited evidence, low demand and shelf space constraints. Ethical tensions around therapeutic vape provision, commercial pressures influencing supply decisions and systemic barriers to counselling including time constraints and lack of remuneration were also identified.

**Conclusions:**

By mid‐2025, therapeutic vapes were available in fewer than half of community pharmacies in South‐East Melbourne, with access skewed towards higher socioeconomic status (SES) areas. Pharmacists faced multiple barriers to the provision of nicotine vaping products and cessation counselling, shaped by commercial determinants and systemic constraints. Further research and regional public health action are needed to support equitable, evidence‐based cessation care in community pharmacy.

**So What:**

Uneven availability of therapeutic vapes across socioeconomic areas risks reinforcing existing inequities in smoking cessation support. Targeted public health strategies are needed to improve equitable access, strengthen pharmacist capacity and ensure implementation of vaping reforms aligns with broader smoking cessation goals.

## 1 Background

Electronic nicotine delivery systems (ENDS), commonly referred to as e‐cigarettes (ECs), are battery‐operated devices that heat nicotine‐containing liquids to produce vapour for inhalation—a practice known as ‘vaping’. In Australia, access to therapeutic vapes is tightly regulated and permitted only for individuals seeking to quit smoking [1]. National cessation guidelines recommend nicotine‐containing therapeutic vapes as a ‘reasonable intervention’ for patients who remain motivated to quit, have previously failed to do so using approved pharmacotherapies, and initiate discussions about vaping with their healthcare provider [2, 3].

Regulation of therapeutic vapes is governed nationally by the Therapeutic Goods Administration (TGA), with some states and territories enacting additional legislation that may further restrict supply. In July 2024, the Australian Government introduced a suite of reforms involving restrictions on ingredients, packaging and flavours of therapeutic vapes, increasing penalties for unlawful supply and establishing a notified list of compliant products [4, 5]. At that time, prior to the introduction of Schedule 3 supply, therapeutic vapes were legally available only via prescription and could not be supplied directly by pharmacists [5]. It was not until October 2024 that the regulatory change permitting pharmacist‐only (Schedule 3) supply of therapeutic vapes was introduced [4, 5]. Importantly, all therapeutic vaping products supplied in Australia remain unregistered medicines, as none are included on the Australian Register of Therapeutic Goods (ARTG), meaning pharmacists supply them under the provisions governing unapproved therapeutic goods [6]. Thus, pharmacists' preparedness to integrate unregistered therapeutic vapes into cessation care under these new reforms remains unclear.

In Victoria, therapeutic vapes may be sold to individuals aged 18 years and over by pharmacists [7]. Therapeutic vapes containing up to 20 mg/mL of nicotine are available without a prescription, while higher concentrations require a prescription from a medical doctor or nurse practitioner [8]. Under Victorian legislation, therapeutic vapes must not be supplied to people under 18 years of age, regardless of prescription status [7]. Products sold in pharmacies must comply with plain packaging requirements and are restricted to mint, menthol or tobacco flavours [8].

Pharmacists have a central role in smoking cessation through the provision of nicotine replacement therapies (NRT), behavioural counselling and referrals to Quitline services [9]. As highly accessible healthcare professionals, they are well‐positioned to deliver brief interventions, monitor progress and collaborate with medical practitioners and cessation services to support smoking cessation [10]. Pharmacist‐led interventions have been shown to significantly increase quit rates, particularly when delivered as part of structured programmes [9]. However, prior to the October 2024 Schedule 3 introduction by the TGA, pharmacists could only supply therapeutic vapes on prescription [11, 12].

The 2024 regulatory changes present both opportunities and challenges for community pharmacies. It remains unclear how many pharmacies will stock therapeutic vapes, their capacity to provide cessation counselling and the extent to which they will adopt evidence‐based strategies incorporating therapeutic vapes. Tobacco and therapeutic vape use are pressing public health concerns in Melbourne's South East metropolitan region, where several local government areas report among the highest smoking and vaping rates in Victoria [13]. The South East Public Health Unit (SEPHU), a key stakeholder in the region, is committed to reducing tobacco and vaping prevalence and mitigating associated health impacts [14]. The SEPHU catchment spans diverse inner, outer and peri‐urban/regional communities, and is home to approximately 1.8 million residents [15]. The 2022 Victorian Smoking and Health Survey reports that several Local Government Areas (LGAs) within the SEPHU region, including Frankston, Port Phillip, Mornington Peninsula, Stonnington and Greater Dandenong, have among the highest smoking and/or vaping rates in metropolitan Melbourne [13].

Given persistent socioeconomic and geographic disparities in smoking rates [16], equitable access to cessation support, including through community pharmacies, remains a critical public health priority. To date, there is limited evidence on how community pharmacies have responded to the 2024 reforms, particularly with respect to stocking therapeutic vapes, and no research has examined spatial equity in access across the SEPHU region. This study aimed to assess the availability of therapeutic vapes in community pharmacies across the SEPHU catchment, and to examine pharmacists' cessation practices and support needs, with a specific focus on the geographic distribution of supply and implications for equitable access.

## 2 Methods

### 2.1 Study Design

We conducted a sequential mixed methods study [17] comprising three components: (1) an audit of therapeutic vape availability in community pharmacies across the South East Public Health Unit (SEPHU) catchment; (2) a survey exploring pharmacists' smoking cessation practices, confidence and service provision and (3) qualitative interviews to contextualise and explain quantitative findings. All three components of the study were conducted between May and July 2025. This design enabled triangulation of data sources to provide a comprehensive understanding of therapeutic vape availability and cessation support in community pharmacy settings.

### 2.2 Setting and Participants

**FIGURE 1:**
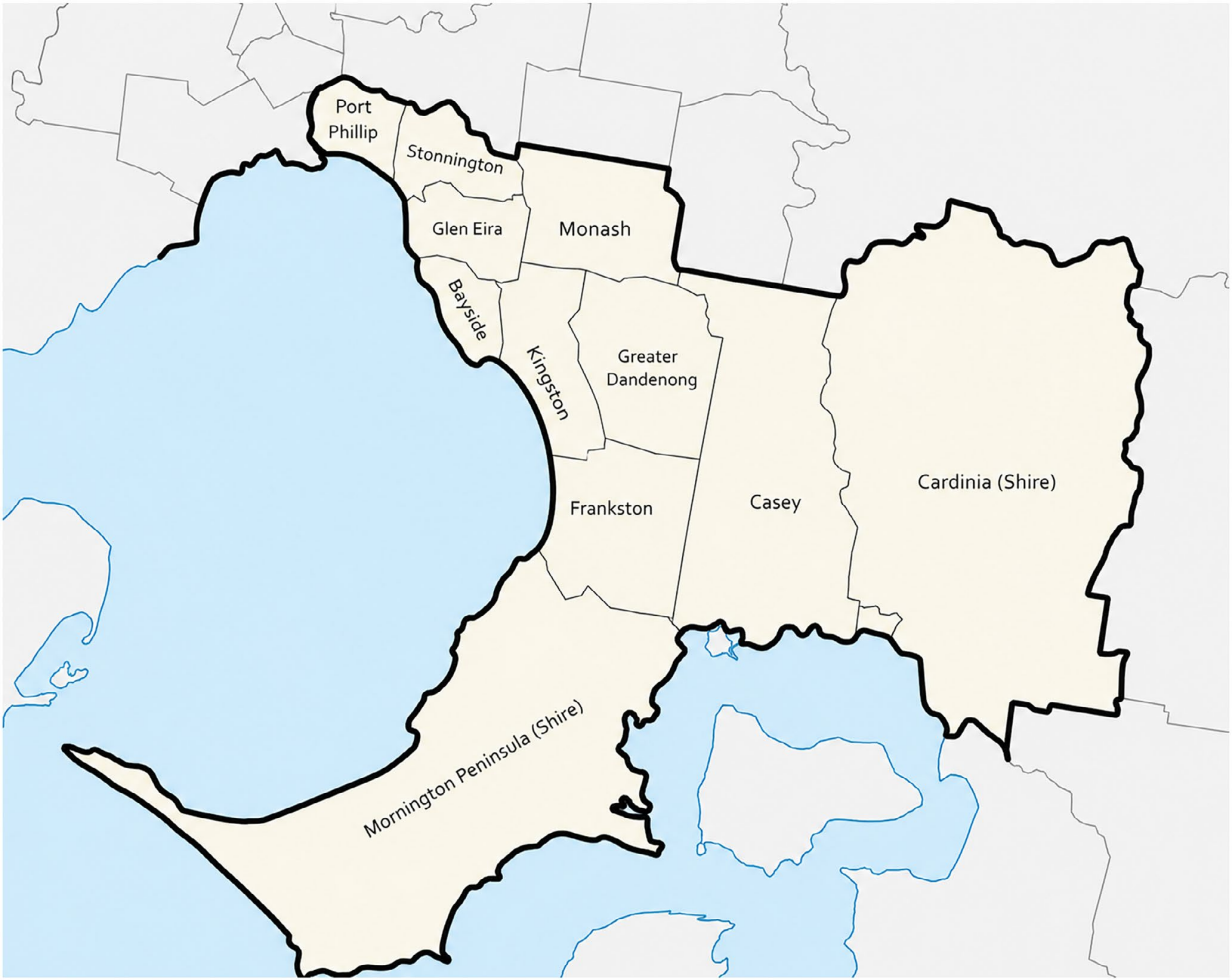
The catchment for the South East Public Health Unit (SEPHU) is located in Melbourne's south east, and encompasses 11 local government areas.

The SEPHU catchment spans inner‐metropolitan LGAs to regional and rural communities, with substantial variation in socioeconomic status (SES), cultural diversity and population density (Figure 1) [15, 18]. Contact details of eligible pharmacies within this catchment were obtained from a national registry of community pharmacies maintained by Monash University's Department of General Practice, cross‐referenced with the Victorian Pharmacy Authority register to include newly established sites. ‘Community pharmacy’ was defined as any registered pharmacy providing walk‐in retail dispensing and counselling services to the public, and included compounding pharmacies. Hospital‐based dispensaries, and online‐only suppliers were excluded.

### 2.3 Recruitment

During the audit, all 418 community pharmacies in the SEPHU catchment were contacted. ‘Pharmacists in charge’ were initially invited to complete an online survey via a QR code sent by fax (*n =* 387) or email (*n =* 31). Pharmacies that did not complete the survey following the fax invitation were subsequently contacted by email if this information was available. Non‐responding pharmacies were followed up three times, after which phone contact was attempted with 390 pharmacies to confirm therapeutic vape availability and identify preferred communication methods. Seven pharmacies were uncontactable despite repeated attempts. The survey link was then re‐sent one final time using each pharmacy's preferred contact method.

### 2.4 Data Collection

#### 2.4.1 Quantitative Component: Audit and Survey

Pharmacies reported whether they dispensed therapeutic vapes and addresses were recorded for mapping. Pharmacists completing the survey (via Qualtrics) provided data on demographics, dispensing of smoking cessation products, counselling services and confidence in delivering the cessation support. The survey was developed in collaboration with SEPHU. We sought further input from QUIT Victoria and the Pharmacy Department of Peninsula health, and pilot tested the survey with nine pharmacists; feedback from QUIT Victoria and seven pharmacists were used to refine the questions. The final survey (see File S1) could be completed between 3 and 5 min. Some survey questions allowed multiple responses, including those relating to training undertaken, flavours dispensed, locations of smoking cessation counselling and resources used to support counselling.

#### 2.4.2 Qualitative Component: Interviews

At the conclusion of the survey, respondents were able to indicate interest in participating in a follow up qualitative interview. Thirteen pharmacists registered interest, and 11 interviews were completed. Suitable times could not be arranged with the remaining two pharmacists during the study period. Interviews were conducted via Zoom by a trained qualitative researcher (I.P.), using a semi‐structured interview guide [19]. Participants received a $40 gift card in recognition of their time. Interviews explored cessation services and experiences with therapeutic vape provision. Audio recordings were transcribed verbatim for analysis using Zoom transcription, and the transcript checked against the audio by IP to ensure accuracy.

### 2.5 Ethics

This research was approved by the Monash University Human Research Ethics Committee, Project ID: 46516. Participants provided consent on beginning the study survey. Verbal consent was obtained from interview participants to audio record and transcribe the interview.

### 2.6 Data Analysis

#### 2.6.1 Quantitative Analysis

Audit and survey data were analysed descriptively using SPSS Version 29. Geographic Information System (GIS) mapping and association analyses were conducted using R. SES was measured using the Australian Bureau of Statistics' Socio‐Economic Indexes for Areas (SEIFA), 2021, specifically the Index of Relative Socio‐economic Advantage and Disadvantage (IRSAD) [20]. Confidence scores were derived from Likert‐scale items (1 = *not at all confidence* to 5 = *very confident*), and mean scores were calculated for each domain. Smoking and vaping prevalence rates for mapping were drawn from the Victorian Smoking and Health Survey 2022 [13]. Incomplete items were excluded listwise; imputation was not used to replace missing items.

Categorical and multiple‐choice variables were summarised using frequencies and proportions. Associations between SES (IRSAD), pharmacy density and therapeutic vape provider density (per 100 000 population), and vaping prevalence were examined using three linear regression models. Firstly, to examine if SES was associated with pharmacy density, a simple regression model was constructed, defining the pharmacy density as the outcome and the SES (IRSAD) as the independent variable. Similarly, in the second model, which was conducted to examine how SES was likely to associate with the local therapeutic vape provider density, the provider density was defined as the outcome, and the SES (IRSAD) was defined as the independent variable. The third multivariant regression model defined the vaping prevalence as the outcome and the provider density as the independent variable, controlling for SES (IRSAD). Scatter plots were generated at the initial phase to explore relationships and assess linearity, normality and homoscedasticity. Spatial analyses were conducted at more than one geographic scale. Spatial distributions of pharmacies by SES (IRSAD) were mapped at both the postal area and LGA levels, as specified in the figure legends for each map.

Statistical significance was set at *p* ≤ 0.05.

#### 2.6.2 Qualitative Analysis

Transcripts were uploaded to QSR NVivo to support data management and coding. Three authors (I.P., N.H. and M.C.) developed a preliminary coding framework, refined through team discussion and iterative questioning. A final coding framework was applied by I.P. and themes were developed following Braun and Clarke's six‐step approach [21]. Rigour was supported through team coding, iterative refinement and documentation of analytic decisions. Qualitative findings were used to aid interpretation and contextualisation of quantitative results, enabling integration across methods.

### 2.7 Results

#### 2.7.1 Pharmacy Audit and Survey Participation

All 418 community pharmacies identified in the SEPHU catchment were included in the audit. Of these, *n* = 174 (41.6%) confirmed availability of therapeutic vapes for smoking cessation, *n* = 224 (53.6%) reported that they did not supply them or were unsure, and *n* = 13 (3.1%) declined to disclose this information. Seven pharmacies (1.7%) were uncontactable after repeated attempts and were classified as ‘uncontactable’.

A total of 70 (16.7%) pharmacists completed the survey with 57 (13.6%) providing complete responses for analysis. Responses were received from pharmacies within each of the 11 LGAs. Most respondents identified as male (*n* = 33/57, 58%), and the remainder identified as female (*n* = 24/57, 42%). Most pharmacists were aged 44 years or younger (*n* = 42/57, 74%), and a large proportion had been practicing for over 10 years (*n* = 34/57, 60%). Pharmacies were mostly independently owned (*n* = 25/57, 44%), or part of a franchise/chain (*n* = 23/57, 40%) with the majority operating small teams of four or fewer pharmacists.

#### 2.7.2 Therapeutic Vape Dispensing Patterns

Among survey respondents, 58% (*n* = 33/57) reported dispensing therapeutic vapes for smoking cessation or management of nicotine dependence. Therapeutic vapes were dispensed between 1–5 times per week, where GP prescription was (*n* = 19/32, 59%) and was not required (*n* = 22/32, 69%). Mint was the most commonly dispensed flavour (*n* = 29/31, 94%), followed by tobacco (*n* = 19/31, 61%) and menthol (*n* = 14/31, 45%).

When asked to rank NRT options, pharmacists recommended nicotine patches, nicotine gum, nicotine lozenges, nicotine oral spray and lastly therapeutic vapes, from most to least often.

#### 2.7.3 Smoking Cessation Services

Among survey respondents, three quarters of pharmacies (*n* = 39/51, 76%) provided smoking cessation counselling, most commonly delivered over the counter (*n* = 27/39, 69%) or in private consultation rooms (*n* = 21/39, 54%). Smoking cessation counselling sessions typically lasted 5–10 min. Staff training was common, with (*n* = 44/52, 85%) reporting some or all pharmacists had completed cessation training, primarily via online modules *(n* = 32/41, 78%) or self‐guided reading or reviewing guidelines (*n* = 22/41, 54%).

### 2.8 Confidence and Counselling Support

Pharmacists expressed high confidence discussing smoking status (mean score: 4.24/5) and cessation strategies (mean score: 4.26/5), but lower confidence in advising patients about use of therapeutic vape products (mean 3.26) and efficacy (mean 3.28). Respondents used supporting materials (*n* = 19/39, 49%), with printed resources (*n* = 14/18, 78%) and websites being most common. All pharmacists reported referring patients to Quitline, GPs or other smoking cessation counsellors.

Follow‐up of patients was variable with only (*n* = 11/38, 29%) of pharmacists reporting they often or always followed up with patients after counselling.

### 2.9 Spatial Distribution of Pharmacies and Therapeutic Vape Availability

**FIGURE 2:**
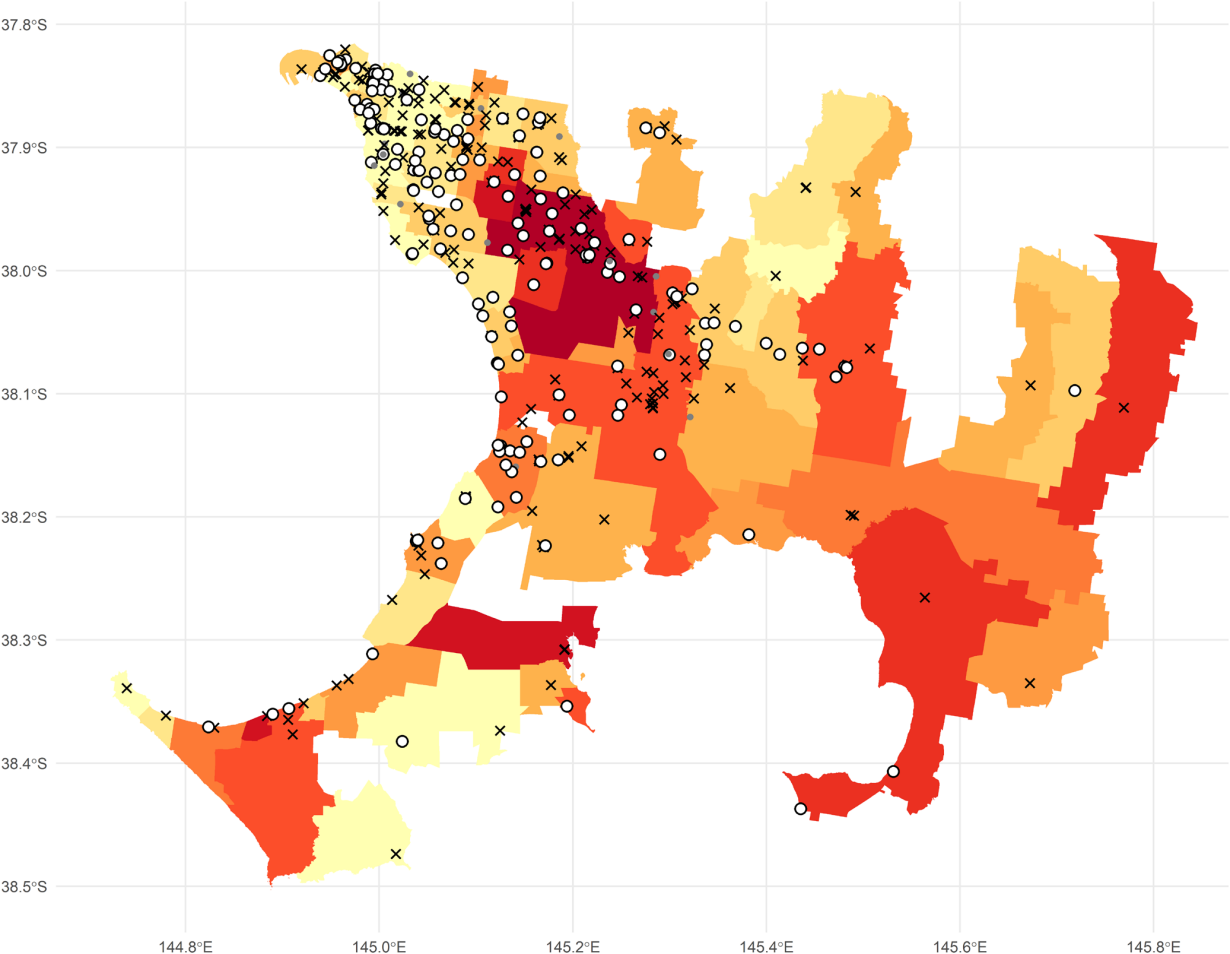
Spatial distribution of pharmacies providing therapeutic vapes for smoking cessation, by socioeconomic status (IRSAD deciles) for postal area, in the South East Public Health Unit (SEPHU) catchment.

**FIGURE 3:**
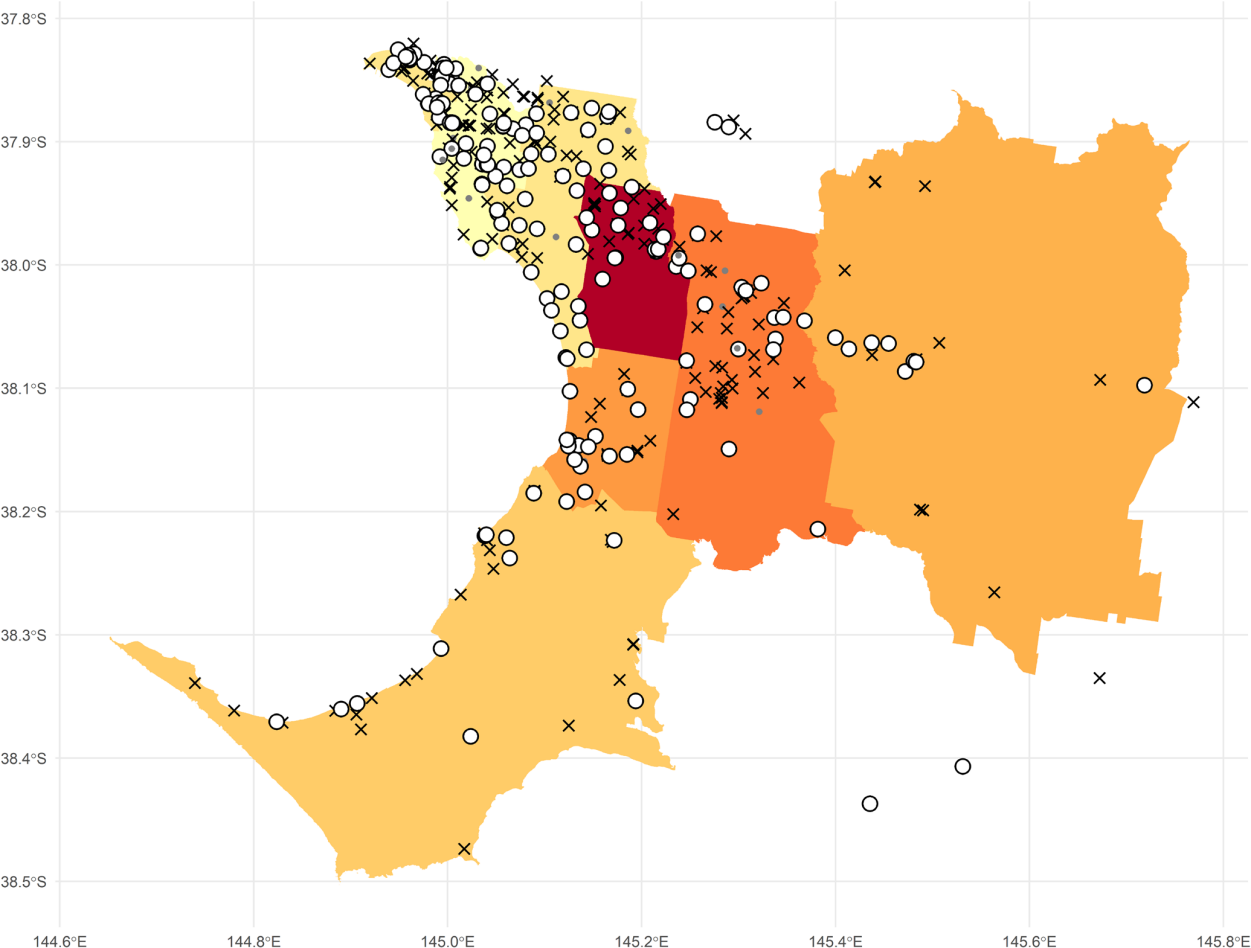
Spatial distribution of pharmacies providing therapeutic vapes for smoking cessation, by socioeconomic status (IRSAD deciles) across local government areas (LGA), in the South East Public Health Unit (SEPHU) catchment.

Geospatial information system (GIS) mapping revealed that the majority of pharmacies supplying therapeutic vapes were disproportionately located in areas of higher SES including Port Phillip, Stonnington, Bayside and Glen Eira (Figures 2 and 3). These areas had both a higher total number of pharmacies and greater therapeutic vape availability. In contrast, lower SES areas such as Greater Dandenong and Casey had both fewer pharmacies per capita and lower therapeutic vape availability, despite reporting high smoking prevalence. This suggests a potential mismatch between need and access, raising equity concerns.

#### 2.9.1 Association With Vaping and Smoking Prevalence

**FIGURE 4:**
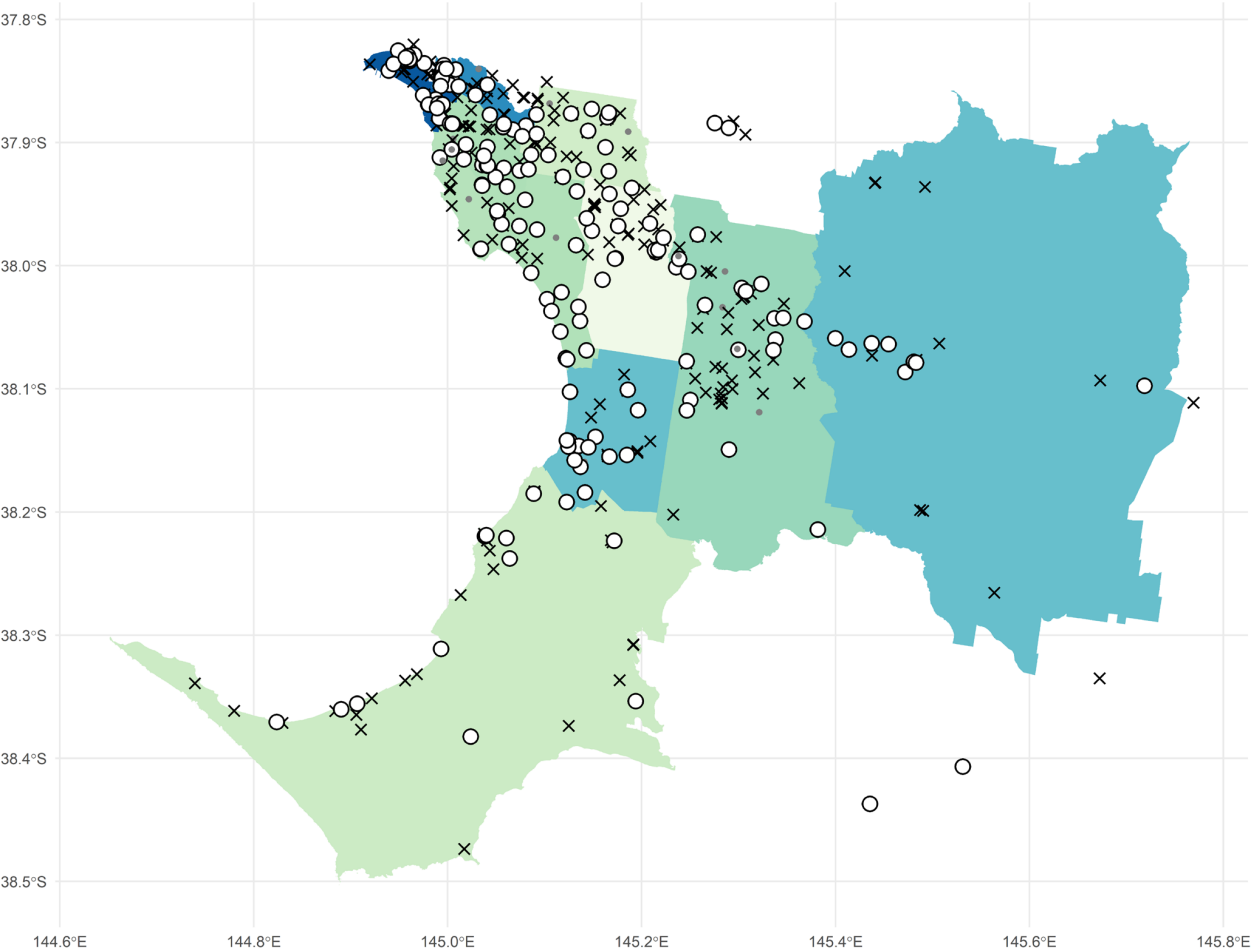
Spatial distribution of pharmacies providing therapeutic vapes for smoking cessation by vaping prevalence (%) [13], across local government areas (LGA), in the South East Public Health Unit (SEPHU) catchment.

**FIGURE 5:**
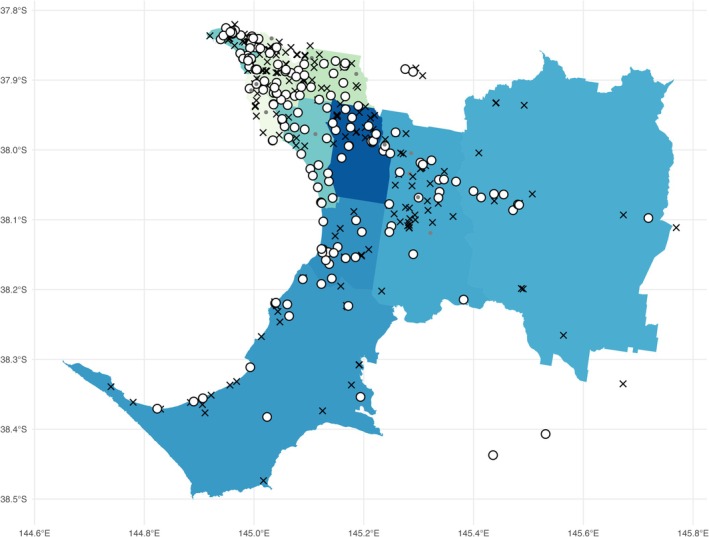
Spatial distribution of pharmacies providing therapeutic vapes for smoking cessation by smoking prevalence (%) [13], across local government areas (LGA) in the South East Public Health Unit (SEPHU) catchment.

Visual inspection of maps indicated that therapeutic vape availability did not consistently align with vaping prevalence. For example, Port Philip and Stonnington had both high therapeutic vape availability, and high vaping rates, while Greater Dandenong had low vaping prevalence despite moderate therapeutic vape access (Figure 4). Smoking prevalence was more evenly distributed, but areas with high SES tended to have greater access to cessation products (Figure 5).

### 2.10 Regression Analysis

Regression analysis showed that for every 10% increase in SES ranking (IRSAD percentile), pharmacy density increased by 1.08 per 100 000 population (95% CI 0.09–2.07; *p* = 0.0324). Nevertheless, this model explained only 4% of the variance of the outcome (adjusted *R* ^2^ = 0.04). There was no statistically significant association between SES and EC‐provider density per 100,00 population (*β* = 0.28; 95% CI: −0.53 to 1.09; *p* = 0.49; adjusted *R* ^2^ = −0.006), suggesting that while pharmacies are more common in advantaged areas, therapeutic vape availability is not uniformly distributed among them (Table 1).

**TABLE 1:** Summary statistics for each local government area.

LGA	SES state ranking	Population	Vaping prevalence (%)	Pharmacy (density)	EC‐provider (density)
Greater Dandenong	1	158 208	4.3	41 (25.92)	16 (10.11)
Casey	5	365 239	6.2	63 (17.25)	18 (4.93)
Frankston	6	139 281	7.1	29 (20.82)	17 (12.21)
Cardinia Shire	7	118 194	7.1	26 (22.00)	12 (10.15)
Mornington Peninsula	8	168 948	5.1	36 (21.31)	11 (6.51)
Kingston	9	158 129	5.7	31 (19.60)	18 (11.38)
Port Phillip	9	101 942	9.1	37 (36.30)	19 (18.64)
Monash	9	190 397	5.0	53 (27.84)	20 (10.50)
Glen Eira	10	148 908	5.8	38 (25.52)	19 (12.76)
Bayside	10	101 306	5.6	26 (25.67)	9 (8.88)
Stonnington	10	104 703	8.3	38 (36.29)	15 (14.33)

*Note:* Density: the number of pharmacies per 100 000 population.

SES state ranking 1 = most disadvantaged, 10 = least disadvantaged.

Smoking and vaping prevalence data sourced from the Victorian Smoking and Health Survey 2022 [13].

**FIGURE 6:**
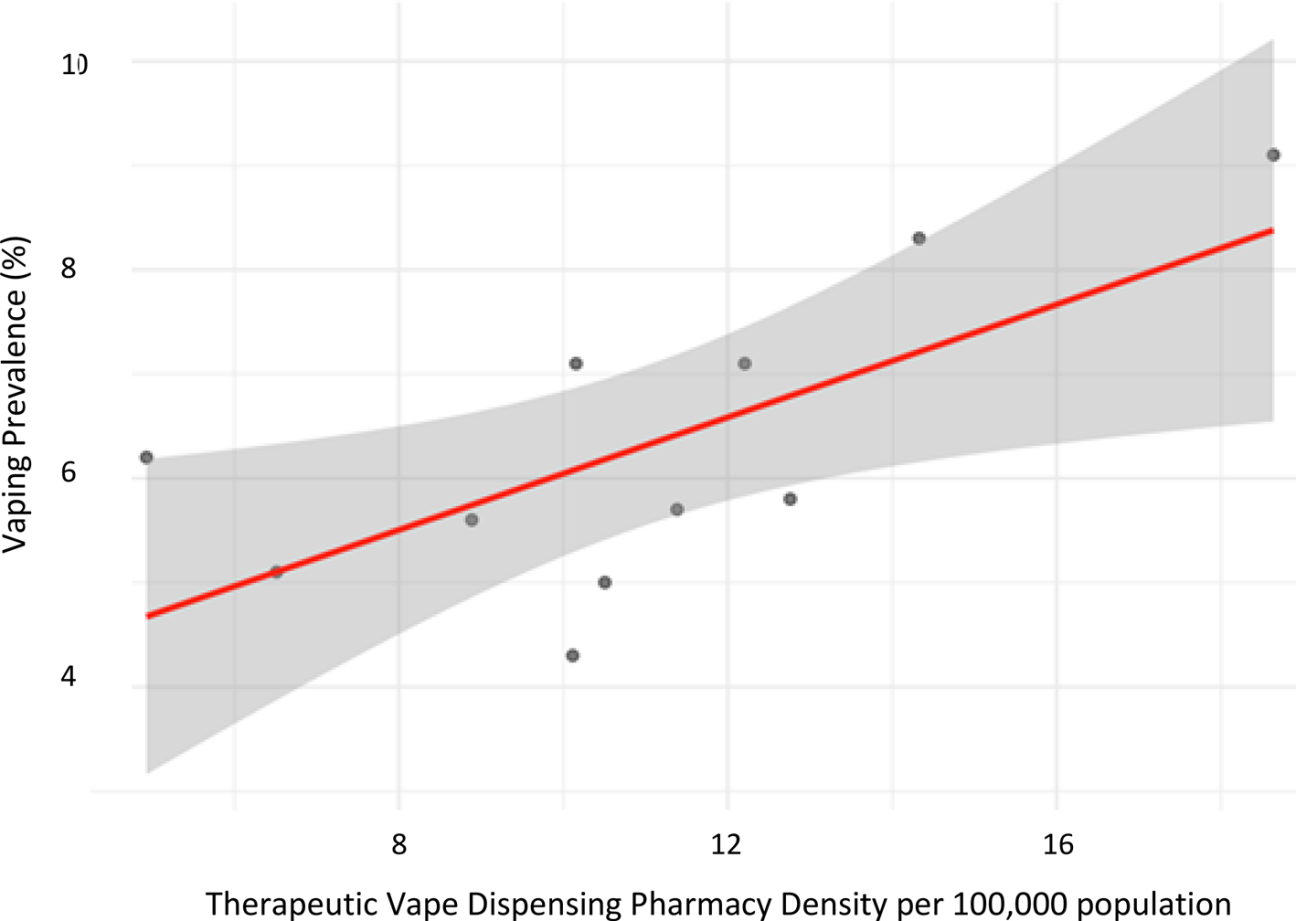
Scatter plot showing the density of pharmacies providing therapeutic vapes per 100 000 population by vaping prevalence in 11 local government areas.

A multivariate model indicated that EC‐provider density was weakly associated with vaping prevalence (Figure 6). For every additional therapeutic vape providing pharmacy per 100 000 population, vaping prevalence increased by 0.25% (95% CI 0.005–0.50, *p* = 0.046).

### 2.11 Qualitative Analysis

Pharmacists who participated in qualitative interviews (*n* = 11) practiced across eight of the 11 catchment LGA's spanning inner urban (*n* = 6: Bayside, Glen Eira, Monash), outer urban (*n* = 3: Frankston, Dandenong, Casey) and peri‐urban/regional areas (*n* = 2: Cardinia, Mornington Peninsula). Four key themes were identified from the interviews with pharmacists reflecting the ethical, commercial, relational and systemic dimensions of therapeutic vape provision for smoking cessation in community pharmacy.

#### 2.11.1 Theme 1: Vaping for Continuation, Not Cessation: Ethical Tensions in Practice

Pharmacists expressed discomfort with supplying therapeutic vapes, citing concerns that patients often sought them for ongoing use (of vaping) rather than for smoking cessation. Many viewed therapeutic vapes as outside their professional scope, preferring existing NRTs such as patches, which were seen as more controlled, affordable and therapeutically sound.

Most pharmacists expressed their reluctance towards therapeutic vape use for smoking cessation, describing an ethical tension with professional beliefs. While pharmacists expressed their comfort in providing smoking cessation services to patients, many felt that the provision of therapeutic vapes fell outside of their professional scope of practice, and they questioned the therapeutic efficacy of this product for smoking cessation.

I have to say the majority of pharmacists do not support ECs… because it takes away what intrinsically we're trying to achieve. I think that this space is not quite our realm, from a professional point of view.(P1, F, > 10 years experience)

Some pharmacists that were interviewed expressed strong support for therapeutic vape use for smoking cessation. They stressed that many pharmacists lacked firsthand experience with nicotine addiction and urged their colleagues to be empathetic and supportive of patients, feeling therapeutic vapes were within their scope of practice.

Most pharmacists have never smoked. They don't know how addictive it is, and it's highly … addictive … We're not tobacconists, but we're there to help people … [pharmacists] need to do their job properly, and they just need to have sympathy or empathy.(P5, F, > 10 years experience)

However, other pharmacists were concerned that patients sought therapeutic vapes for ongoing use rather than cessation, undermining the therapeutic intent of supply. They expressed that the provision of therapeutic vapes in pharmacy made them feel like ‘tobacconists’ rather than clinicians, and thus went against pharmaceutical supply principles.

A lot of the times … when [customers] come in … [and request therapeutic vapes], very little percentage are even interested in coming off it. They're there for the supply … it becomes a behaviour issue … we all felt it became more like a supply after that, and it just completely took away what pharmacy was for us.(P1, F, > 10 years experience)

Pharmacists noted that those presenting for the supply of therapeutic vapes were often doing so to ensure device safety, as patients wanted peace of mind that their device is coming from a reputable source.

They want to use a safer device … to transition from their smoke store … to one that … while not technically approved … the ingredients are known, and it's coming from a reputable source.(P2, M, 0–5 years experience)

One pharmacist emphasised the importance of reframing vaping as smoking cessation in conversations with patients, suggesting this would influence patients' intentions to use therapeutic vapes for smoking cessation.

We do not call it vaping. We call it smoking cessation, because that's what it is.(P5, F, > 10 years experience)

Ultimately, most pharmacists agreed that nicotine patches remain the gold standard product for smoking cessation. Pharmacists felt confident supplying this product due to its steady and more easily controlled delivery, noting also that it is more affordable for patients to purchase.

I have to say the patches are always going to be gold standard for me in a … community setting … purely because it's got a steady delivery. It's controlled, you know where you're at with the 3 steps … the prices have come down significantly … it's easier to buy a week each time … They can touch it. They can feel it … it gives them reassurance … acts as a reminder that they are wearing something that's giving them nicotine.(P1, F, > 10 years experience)

#### 2.11.2 Theme 2: Between Care and Commerce: Navigating Financial and Regulatory Pressures

Pharmacists described tensions between clinical care and commercial realities. While some acknowledged the potential financial benefits of therapeutic vape sales, others highlighted the lack of remuneration for smoking cessation counselling as a barrier to sustained engagement.

Pharmacists described the time (often 10–15 min) they felt was needed to provide meaningful smoking cessation counselling to patients as not practical in a busy, resource constrained pharmacy setting.

I think [what needs] to be considered is the time that the pharmacists take with the customer … it should be a…paid service that we can claim … so we can spend more time … if it's not [benefiting] the pharmacy … [it would] definitely [be] discouraging to do it.(P4, F, 6–10 years experience)

Pharmacists described challenges in choosing which brand of therapeutic vape to supply in their pharmacy. Pharmacists noted that illicit supply of therapeutic vapes often involves disposable devices, whereas the therapeutic vapes in pharmacies have pods that are device‐specific. Pharmacists then had to consider whether they wanted an exclusive brand, where patients would have to return to their pharmacy to purchase these pods, or whether they wanted to stock a brand more readily available in other pharmacies and thus their customer base.

Each device has its own pod … so when you stock [a specific brand] you need to make this easy to access for the patients. Because if you compare the alternative, it is so easy to access, you walk into vape shops and just get it [they're] all one size fits all, because they're disposables, it's sort of juggling two sides of the puzzle from a business perspective. We'd like an exclusive brand, people return to us for pods but then also from capturing an audience, we want the most readily available.(P2, M, 0–5 years experience)

I found talking about flavours … is a thing for the customer like ‘Oh do you have this and that?’ And we're sitting down like ‘Oh, [would] you like to try it out?’ … [It's not] very professional.(P4, F, 6–10 years experience)

Another barrier to providing meaningful smoking cessation support using therapeutic vapes was the lack of visibility and knowledge regarding patients' previous supply history and limited communication between GPs and pharmacists. Pharmacists feared that when supplying via the SAS‐C pathway, which allows health practitioners to access unapproved therapeutic goods without needed prior TGA approval and without access to previous supply records, they were forced to rely solely on patient self‐reporting, which is unreliable and could result in misuse, thus undermining the service's clinical value [22].

We [could] do it as a project stop [online platform for pharmacists to determine therapeutic needs of patient] also, you can't see previous supply as well. Let's say, if the customer came in and I asked, ‘how often do you go through this?’ He could say anything … because I don't know previous supplies, when it happened, how many he got. It's really essential to know.(P4, F, 6–10 years experience)

These commercial and regulatory pressures shaped not only pharmacists' supply decisions, but also their capacity to engage meaningfully with patients—a tension explored in the next theme.

#### 2.11.3 Theme 3: From Convenience to Connection: Community Pharmacy as a Trusted Setting for Behaviour Change

Pharmacists viewed community pharmacy as an accessible and trusted setting for smoking cessation support. Familiarity with patients enabled informal, patient‐led conversations that could evolve into meaningful behaviour change discussions.

They described strong rapport with regular patients, often built through ongoing medication supply. These ongoing interactions, which sometimes extended to patients' family members, often involved informal conversations initiated by patients. Pharmacists believed that this familiarity fostered a supportive space that could lead to behaviour change conversations, such as smoking cessation, when patients felt ready to engage.

It's so easy for them [customers] to come in, particularly when they know us really well. A lot of them already have other regular medications. Some of them come with their children that have taken up smoking because they're following on with the parents. They just want to have a chat or they start disclosing other social implications like money, rent, relationship, breakdown … whatever it is, and then suddenly you become a counsellor, you're no longer a pharmacist. You become an information hub.(P1, F, > 10 years experience)

Pharmacists emphasised the importance of patient‐centred communication, and delivering smoking cessation counselling in a way that was patient‐led, thus respecting individual readiness and pace. Counselling was often initiated by the patient, and pharmacists tailored their support to align with each patient's preferences and progress.

This is a journey we take together [patient/pharmacist] … This is your journey. You make the steps. I support you the way we're going.(P5, F, > 10 years experience)

When conducting smoking cessation counselling, pharmacists often engaged in goal‐oriented conversations in order to tailor both the counselling approach and product selection to each individual patient. These conversations often aimed to identify patients' motivations, readiness to quit and smoking patterns and triggers in order to identify the most appropriate therapeutic support.

What are you hoping to achieve? When do you want to quit? Sort of setting your start date? What your pattern currently is? How much you smoke, are you a first time [quitter]? Sort of first thing in the morning, more in the evening, like sort of the pattern, triggers to try and determine the most appropriate product. And then, once you've got a good picture of what their goals are, and what their patterns of behaviour are, then we'd go from there.(P7, F, > 10 years experience)

#### 2.11.4 Theme 4: Strengthening the System: The Needs of Pharmacists for Effective Smoking Cessation Practice

Pharmacists identified several system‐level improvements that could enhance their capability to provide smoking cessation care. These included clearer dose reduction pathways to guide therapeutic vape discontinuation, ongoing education and improved communication with GPs.

Some pharmacists acknowledged that though therapeutic vapes are promoted as a smoking cessation aid, the ultimate therapeutic goal is for patients to discontinue therapeutic vape use entirely. To facilitate this transition, pharmacists voiced their need for clearer dose reduction pathways to facilitate smoking and therapeutic vape cessation.

I suppose the ultimate aim is coming off them. I think there needs to be more support on how to come off therapeutic vapes as well. More research and more evidence showing guidelines on how to come off vapes when we do transition to cessation.(P6, F, > 10 years experience)

One pharmacist described their use of a manufacturer‐developed programme to calculate dosages and generate a cessation plan based on patients' smoking and/or vaping habits. While the pharmacist acknowledged the resource's usefulness, its commercial origins suggest the need for an independent, evidence‐based dose reduction pathway to support both smoking and therapeutic vape cessation.

[The] program [I use … you're] able to put in how much [the patient] is vaping, or how much [the patient] is smoking, it'll give me an idea of what [they're] spending a year on cigarettes, and if [they] move to vaping what [they're] going to save, and go through the procedure on how I expect them to move, going forward on the vapes. It's even got all the brands of the cigarettes, how much [they're] smoking. Obviously, it's company driven, but as a resource it's really good.(P5, F, > 10 years experience)

Some pharmacists also emphasised the importance of continuing education to improve the quality of smoking cessation counselling. Though they acknowledged the time constraints inherent in their practice, pharmacists felt that semi‐regular, in‐person educational visits would be beneficial to reinforce existing knowledge and introduce updated guidance.

More frequent continuing education would be good. I know we're all time poor, and I know we have access to [Continuing Professional Development] Modules, and we can. But the reality is, every pharmacist doesn't have much time. I learned so much from my reps when they come in the store. A course would be really good and continuity every 6 months would be good.(P11, F, > 10 years experience)

Pharmacists also expressed a desire for improved communication between pharmacists and GPs in order to enhance smoking cessation care and coordination. Pharmacists noted the importance of communication in order to determine appropriate dosages and to ensure that prescribed products are readily available in pharmacy.

In terms of the logistic side of things, with the GP, a link would definitely help. If GPs are really intent on prescribing them, if GPs do consult with their pharmacist more so that they can give the right prescription or a prescription with a product readily available, then, yeah, that would be nice.(P6, F, > 10 years)

## 3 Discussion

This study provides a timely examination of therapeutic vape availability and pharmacists' perspectives following the 2024 regulatory reforms enabling pharmacy‐based supply in Australia. Using a mixed‐methods approach, we identified variability in therapeutic vape availability across South‐East Melbourne, with access skewed towards higher socioeconomic areas. While pharmacists are well positioned to support smoking cessation, our findings highlight persistent barriers to both product provision and counselling that were shaped by ethical concerns, commercial pressures and systemic constraints. This uneven distribution raises concerns about equitable access to regulated cessation support.

Pharmacy is often regarded as a highly accessible health care service for Australians. According to Australian Health Practitioner Regulation Agency (AHPRA) reports, there were approximately 132.7 pharmacists per 100 000 population in Victoria in 2022, with most working in community pharmacy. But pharmacists are not distributed evenly across metropolitan, regional and rural areas, with a greater proportion working in metropolitan regions. This distribution is seen in our data as well, but beyond this, most pharmacies clustered not just in metropolitan regions, but in high SES suburbs of metropolitan areas. This pattern may inadvertently reinforce existing health disparities, particularly in areas with high smoking prevalence but limited access to cessation support.

In south‐east Victoria, vaping prevalence is greatest in high SES suburbs including Port Phillip and Stonnington, while smoking prevalence tends to be greater in low SES suburbs such as Dandenong [13]. Our geospatial analyses showed a corresponding trend between density of pharmacies with therapeutic vape availability and vaping prevalence. This trend was not seen in regions where smoking prevalence is higher. Further research and surveillance are needed to distinguish therapeutic use from recreational uptake, particularly in high‐access regions. This could determine whether the greater prevalence of vaping in the regions with greater density of pharmacies with therapeutic vapes available reflects therapeutic vape users switching from cigarettes to therapeutic vapes for smoking cessation, or whether therapeutic vapes are being used in these areas for non‐therapeutic reasons. Notably, interpretation of these findings should also consider the time point at which this data was collected, which occurred during the early implementation phase of the 2024 regulatory reforms. At the time of our survey, community pharmacy supply of therapeutic vapes was newly established, and distributor stock was limited [23]. Further, pharmacist education and professional guidance, particularly through the Pharmaceutical Society of Australia (PSA), were in the process of being rolled out [10].

Pharmacists expressed concerns regarding the therapeutic efficacy and role of therapeutic vapes in smoking cessation. These findings were consistent with reports from other pharmacists in NSW [24] and are consistent with findings from other health professional groups such as GPs [25]. These concerns reflect broader uncertainty in the evidence base, with ongoing debate about the efficacy and safety of therapeutic vapes as cessation tools [26]. Smoking cessation guidelines in Australia emphasise the role of therapeutic vapes in smoking cessation as secondary to other established treatments including behavioural counselling and nicotine replacement therapy [3]. There is concern among GPs and pharmacists about the proliferation of therapeutic vapes for purposes other than smoking cessation, and these concerns were detected in qualitative interviews with pharmacists in this study. This finding has important implications for clinical practice, highlighting the need for clear guidance on the appropriate use of therapeutic vapes for smoking cessation, and in particular, the dose reduction of nicotine in therapeutic vapes as part of a legitimate smoking cessation plan.

Our findings, and in particular the ethical tensions described by some pharmacists, can be understood through a commercial determinants of health lens, which highlights how market forces and profit motives shape health service delivery and influence equity [27]. These ethical tensions are not new in pharmacy [28]. Pharmacists in our study described tensions between their clinical role and commercial pressures, including decisions about which therapeutic vape brands to stock, how to respond to patient demand for flavours, and whether to invest time in non‐remunerated counselling. The emergence of online therapeutic vape suppliers further complicates this landscape, introducing new commercial actors with limited accountability for therapeutic outcomes. These dynamics raise important questions about how commercial interests intersect with public health objectives in community pharmacy settings.

Another important consideration is the burden with supplying unregistered therapeutic vaping products in community pharmacy. As therapeutic vaping products are not included on the ARTG, pharmacists must supply them under the regulatory provisions for unapproved therapeutic goods [6]. This classification introduces added professional responsibility. Pharmacists in this study described their hesitation with supplying therapeutic vapes, which may well reflect these regulatory and evidentiary uncertainties, as opposed to opposition to their use for smoking cessation support. Further, supply of therapeutic vapes also increases their workload, with pharmacists having to invest additional time in documentation, and of course, counselling, without corresponding remuneration [29]. These demands occur within already time‐constrained community pharmacy environments, potentially contributing to variability in uptake of therapeutic vape supply across community pharmacies seen in our analysis. Addressing these burdens will be critical to supporting consistent and equitable implementation of the 2024 reforms.

Pharmacists in Australia play an important role in providing smoking cessation support in local communities. While intensive specialist support services (e.g., Quit Line) may have higher quit rates than one‐to‐one services provided by pharmacies, pharmacy services treat many more people who smoke and are cost effective [30]. Similarly, more intensive structured care given by community pharmacy staff probably helps more people to quit smoking than less intensive support to quit [9]. The pharmacists in our study expressed confidence in their capabilities and in particular, the utilisation of NRTs to support smoking cessation, a treatment most of the pharmacists in our study considered to be the gold standard. While guidelines for pharmacists that incorporate support for smoking cessation using therapeutic vapes are available [3, 10], continued work to promote these guidelines and provide training in their use would be helpful. In particular, guidance for the dose reduction of nicotine in therapeutic vapes was desired so that patients were not simply substituting therapeutic vapes for cigarettes, but were being supported to address and resolve nicotine dependence.

Identifying patient counselling barriers is an important intervention for providing effective patient counselling. In pharmacy, these barriers commonly include time constraints, lack of privacy or confidentiality, insufficient patient information, difficulty in accessing pharmacist or drug information promptly, language barriers or poor communication skills [31]. There were both commercial considerations as well as system barriers, such as ensuring there were sufficient pharmacists available to support other patients during counselling sessions. Further, smoking cessation counselling is not renumerated in pharmacy, and so there is a disincentive for pharmacists to provide more engaged advice and support. Addressing these barriers may require structural enablers such as dedicated consultation spaces, integrated digital tools and funding for counselling services.

### 3.1 Strengths and Limitations

A key strength of this study is its grounding in local public health priorities. The research question was identified in collaboration with the South East Public Health Unit (SEPHU), a key stakeholder in this region. The study, design, data collection and interpretation were informed by SEPHU's input, enhancing the relevance and translational potential of the findings.

The research team brought together diverse expertise, including clinicians, pharmacists, public health researchers and medical students, enabling a multi‐disciplinary lens on smoking cessation and therapeutic vape provision. The qualitative component was strengthened by triple coding and iterative thematic analysis, supporting rigour and credibility.

The mixed methods design allowed for integration of quantitative and qualitative data, providing both breadth and depth of insight. Spatial mapping added a systems‐level perspective, highlighting geographic and socioeconomic patterns in access that may otherwise be overlooked.

However, several limitations should be noted. The study was conducted in a single region (South‐East Melbourne), and as states adopt increasingly divergent approaches to therapeutic vape regulation, findings may not be generalisable to other jurisdictions. Additionally, the study did not include emerging ‘online pharmacies’ that supply therapeutic vapes. These platforms represent both a commercial competitor to community pharmacies and raise important questions about the quality and accessibility of smoking cessation counselling in digital environments. Finally, survey data were self‐reported and may be subject to recall or social desirability bias; while responses were received from pharmacies in all LGAs within the SEPHU catchment, anonymisation to protect participants given the sensitivity of questions relating to clinical capability precluded finer‐grained geographic analysis.

The survey response rate was modest, and the qualitative sample was relatively small, limiting diversity in perspectives captured. Nonetheless, the mapping and qualitative data were rich and meaningful, enabling in‐depth exploration of pharmacists' experiences. The integration of methods strengthens the explanatory power of the findings. Future research could extend this work to other regions, incorporate patient perspectives and examine the impact of targeted interventions to support equitable access and pharmacist capability.

Future research should focus on longitudinal tracking of therapeutic vape supply in community pharmacy, along with repeated spatial analyses to assess changes in geographic and socioeconomic equity over time. Qualitative studies examining pharmacist and patient experiences as the reforms mature will also be valuable to inform policy and practice.

## 4 Conclusion

Regional public health authorities are well‐positioned to respond to the equity and practice gaps identified in this study. Potential actions include supporting pharmacist training in smoking cessation counselling, facilitating partnerships between pharmacies and other community stakeholders, and exploring mechanisms to improve access to cessation support, particularly in lower‐SES postcodes, where pharmacy density is lower. At a regulatory level, the TGA may play an important role in monitoring and providing guidance on therapeutic vape supply, including emerging online channels, in order to ensure safe and appropriate therapeutic use. Further research is needed to understand and monitor online therapeutic vape supply and the impact of this on therapeutic engagement. There is a need to ensure pharmacy practice aligns with broader public health goals of preventing the proliferation of vaping, while supporting options for smoking cessation.

Therapeutic vapes are unevenly accessible across South‐East Melbourne, with availability skewed towards higher socioeconomic areas raising equity concerns. This study highlights the complex and often conflicting roles pharmacists navigate in supporting smoking cessation, shaped by ethical tensions, commercial pressures and systemic constraints. While pharmacists view community pharmacy as a trusted and accessible setting for cessation support, barriers such as time constraints, insufficient training and unclear guidance continue to affect both product provision and counselling quality. Further research is needed to clarify pharmacists' roles, strengthen system supports and evaluate the effectiveness of therapeutic vapes in community‐based cessation care.

## Funding

The authors have nothing to report.

## Ethics Statement

The Monash University Human Research Ethics Committee (Project #46516) granted ethical approval for this study on 26/05/2025. An amendment to the approved protocol was subsequently granted on 04/08/2025 to address lower‐than‐anticipated survey response rates and to support completion of the quantitative component of the study. The amendment permitted additional contact with community pharmacies using publicly available contact details to confirm the availability of therapeutic vaping products, including follow‐up telephone contact and use of a standardised script. The amendment also allowed further contact to facilitate survey participation by pharmacists.

## Conflicts of Interest

The authors declare no conflicts of interest.

## Supporting information


**File S1:** Study survey.

## Data Availability

The data that support the findings of this study are available on request from the corresponding author. The data are not publicly available due to privacy or ethical restrictions.
