# Supplementary material for: Mapping Access to Nicotine Vaping Products for Smoking Cessation: Pharmacist Needs and Practice Implications in South‐East Melbourne
Source: Health Promot J Austr. 2026 Jul 3;37(3):e70213. doi: 10.1002/hpja.70213 (PMC13329802; doi:10.1002/hpja.70213)
Supplement: Supplementary file 1 — File S1: Study survey. [file HPJA-37-0-s001.docx]

Supplemental File 1: Study Survey

**Assessing the regional accessibility of e-cigarettes for smoking cessation at community pharmacies in South East Melbourne (Project ID: 46516)**

Thank you for your interest in participating in the study.

The survey aims to explore regional availability of nicotine vaping products as smoking cessation aids and delivery of smoking cessation counselling services at community pharmacies.

This research is being undertaken by researchers from the School of Public Health and Preventive Medicine at Monash University, together with partners at the South East Public Health Unit at Monash Health.

All data collected will be kept securely at Monash University, participation in this survey is voluntary.

The survey is expected to take approximately 3 minutes to complete. At the end of the survey there is a chance to express interest to participate in a follow-up qualitative interview. If you are interviewed, a gift card of $40 will be provided for your time.

You can read the **explanatory statement** of the study here.

Proceed to the survey to indicate your consent to take part in the study.

End of Block: Introduction

Start of Block: Background and Description Information

Please record the address of your pharmacy below:

________________________________________________________________

________________________________________________________________

________________________________________________________________

________________________________________________________________

________________________________________________________________

This survey is intended to be completed by the Pharmacist in Charge. Please confirm you hold this role at this pharmacy:

- Yes (4)
- No (5)

Which age group are you in?

- Under 35 (1)
- 35-44 (2)
- 45-54 (3)
- 55-64 (4)
- Over 64 (5)

What is your gender?

- Woman (11)
- Man (12)
- Non-binary (13)
- Prefer not to say (14)

How many years have you been practicing as a pharmacist?

- 0-5 years (1)
- 6-10 years (4)
- Over 10 years (5)

| Page Break |  |
| --- | --- |

Please select which model best describes your pharmacy:

- Independently owned (6)
- Partnership (7)
- Franchise/Chain (8)
- Other (Please specify) (9) __________________________________________________

Including yourself, how many pharmacists do you have working in your pharmacy?

- 1-2 (2)
- 3-4 (4)
- 5-6 (5)
- 7 or more (6)

How many professional staff (other than pharmacists, e.g. pharmacy technicians/assistants) do you have working in your pharmacy?

- 1-2 (2)
- 3-4 (3)
- 5-6 (5)
- 7 or more (6)

End of Block: Background and Description Information

Start of Block: Dispensing practice of e-cigarettes

Is your pharmacy dispensing therapeutic vapes as a treatment option for smoking cessation or management of nicotine dependence?

- Yes (1)
- No (2)
- Unsure (3)

Display this question:

If Is your pharmacy dispensing therapeutic vapes as a treatment option for smoking cessation or mana... = Yes

How often do you dispense therapeutic vapes from a prescription **from GPs**?

- 0 times a week (27)
- 1-5 times a week (28)
- 6-10 times a week (29)
- 11-15 times a week (30)
- More than 15 times a week (31)

Display this question:

If Is your pharmacy dispensing therapeutic vapes as a treatment option for smoking cessation or mana... = Yes

How often do you dispense therapeutic vapes in cases where a prescription is **NOT** required (**as Schedule 3**)?

- 0 times a week (17)
- 1-5 times a week (18)
- 6-10 times a week (19)
- 11-15 times a week (20)
- More than 15 times a week (21)

| Page Break |  |
| --- | --- |

Display this question:

If Is your pharmacy dispensing therapeutic vapes as a treatment option for smoking cessation or mana... = Yes

What flavours of therapeutic vapes are you currently dispensing? (Select all that apply)

- Mint (1)
- Menthol (2)
- Tobacco (3)
- Other (Please specify) (4) __________________________________________________

Display this question:

If Is your pharmacy dispensing therapeutic vapes as a treatment option for smoking cessation or mana... = Yes

In an average week, how often do you dispense the following therapeutic vapes at your pharmacy?

|  | Not available (1) | 0-3 times (2) | 4-6 times (3) | 7-10 times (4) | More than 10 times (5) |
| --- | --- | --- | --- | --- | --- |
| Nicotine >20 mg/mL (2) |  |  |  |  |  |
| Nicotine ≤20 mg/mL (5) |  |  |  |  |  |
| Nicotine-free (6) |  |  |  |  |  |

| Page Break |  |
| --- | --- |

In an average week, how often do you dispense the following prescription medications at your pharmacy?

|  | 0-3 times (4) | 4-6 times (5) | 7-10 times (8) | More than 10 times (9) |
| --- | --- | --- | --- | --- |
| Varenicline (Champix) (1) |  |  |  |  |
| Bupropion (Zyban) (2) |  |  |  |  |

Please rank from most (1) to least (5) often that you recommend the following nicotine replacement therapy options to your patients for smoking cessation:

______ Nicotine patches (14)

______ Nicotine gum (15)

______ Nicotine lozenges (16)

______ Nicotine oral spray (17)

______ Nicotine vaping products (e-cigarettes) (18)

End of Block: Dispensing practice of e-cigarettes

Start of Block: Smoking cessation counselling

Have your pharmacy staff undertaken any training in smoking cessation?

- Yes, all staff (12)
- Yes, only some staff (13)
- No (14)
- Unsure (15)

Display this question:

If Have your pharmacy staff undertaken any training in smoking cessation? = Yes, all staff

Or Have your pharmacy staff undertaken any training in smoking cessation? = Yes, only some staff

And If

Have your pharmacy staff undertaken any training in smoking cessation? != No

Or Have your pharmacy staff undertaken any training in smoking cessation? != Unsure

Which staff have been included in the training for smoking cessation at your pharmacy?

- Pharmacists (1)
- Pharmacy technicians (2)
- Pharmacy assistants (3)

Display this question:

If Have your pharmacy staff undertaken any training in smoking cessation? = Yes, all staff

Or Have your pharmacy staff undertaken any training in smoking cessation? = Yes, only some staff

And If

Have your pharmacy staff undertaken any training in smoking cessation? != No

Or Have your pharmacy staff undertaken any training in smoking cessation? != Unsure

What training have your staff undertaken for smoking cessation? (Select all that apply)

- Online training modules (6)
- Webinars/workshops (7)
- Self guided reading or reviewed guidelines (4)
- Other (Please specify) (5) __________________________________________________

Display this question:

If Have your pharmacy staff undertaken any training in smoking cessation? = Yes, all staff

Or Have your pharmacy staff undertaken any training in smoking cessation? = Yes, only some staff

And If

Have your pharmacy staff undertaken any training in smoking cessation? != No

Or Have your pharmacy staff undertaken any training in smoking cessation? != Unsure

Which organisation(s) are you accessing the training materials from?

________________________________________________________________

________________________________________________________________

________________________________________________________________

________________________________________________________________

________________________________________________________________

| Page Break |  |
| --- | --- |

Are you providing smoking cessation counselling at your pharmacy?

- Yes (1)
- No (2)
- Unsure (3)

Display this question:

If Are you providing smoking cessation counselling at your pharmacy? = Yes

Where are you conducting these smoking cessation counselling services in your pharmacy? (Select all that apply)

- In a private consultation room/area (5)
- Over the counter (1)
- At a desk (2)
- Behind a partition (3)
- In pharmacy aisles (4)
- Other (Please specify) (6) __________________________________________________

Display this question:

If Are you providing smoking cessation counselling at your pharmacy? = Yes

How long does the smoking cessation counselling usually take?

- Under 5 minutes (1)
- 5 to under 10 minutes (4)
- 10 to 20 minutes (6)
- Over 20 minutes (7)

Display this question:

If Are you providing smoking cessation counselling at your pharmacy? = Yes

Are you utilising any resources or supporting materials to assist in your smoking cessation counselling?

- Yes (1)
- No (2)
- Unsure (3)

Display this question:

If Are you utilising any resources or supporting materials to assist in your smoking cessation couns... = Yes

What resources or supporting materials are you using to assist you in your smoking cessation counselling? (Select all that apply)

- Printed materials (7)
- Websites (5)
- Audiovisual aids (4)
- Other (Please specify) (6) __________________________________________________

Display this question:

If Are you utilising any resources or supporting materials to assist in your smoking cessation couns... = Yes

Which organisation(s) are you accessing these resources from?

________________________________________________________________

________________________________________________________________

________________________________________________________________

________________________________________________________________

________________________________________________________________

| Page Break |  |
| --- | --- |

Which of the following services do you actively recommend to your patients for smoking cessation?

|  | Always (1) | Often (3) | Sometimes (4) | Never (5) |
| --- | --- | --- | --- | --- |
| Quitline (6) |  |  |  |  |
| General Practitioners (7) |  |  |  |  |
| Smoking cessation counsellors (other than Quitline) (8) |  |  |  |  |
| Other (Please specify) (9) |  |  |  |  |

Display this question:

If Are you providing smoking cessation counselling at your pharmacy? = Yes

Do you follow up with patients after providing smoking cessation counselling?

- Always (7)
- Often (8)
- Sometimes (9)
- Never (10)

End of Block: Smoking cessation counselling

Start of Block: Level of confidence in smoking cessation

Thinking about the use of nicotine vaping products (i.e. e-cigarettes) for smoking cessation, how would you describe your level of confidence in the following?

|  | Not at all confident (1) | Neither confident nor not confident (3) | Somewhat confident (4) | Very confident (5) |
| --- | --- | --- | --- | --- |
| Knowledge about the efficacy of e-cigarettes for smoking cessation (1) |  |  |  |  |
| Knowledge about current regulatory requirements on dispensing e-cigarettes (2) |  |  |  |  |
| Knowledge about referral to smoking cessation support services (3) |  |  |  |  |
| Ability to talk to patients about their smoking status (4) |  |  |  |  |
| Ability to talk to patients about proper use of e-cigarette products (5) |  |  |  |  |
| Ability to answer questions about smoking cessation (6) |  |  |  |  |

End of Block: Level of confidence in smoking cessation
